# Low-grade albuminuria is associated with early but not late carotid atherosclerotic lesions in community-based patients with type 2 diabetes

**DOI:** 10.1186/1475-2840-12-110

**Published:** 2013-07-24

**Authors:** Mei-Fang Li, Yin-Fang Tu, Lian-Xi Li, Jun-Xi Lu, Xue-Hong Dong, Li-Bo Yu, Rong Zhang, Yu-Qian Bao, Wei-Ping Jia, Ren-Ming Hu

**Affiliations:** 1 Department of Endocrinology and Metabolism, Shanghai Jiao Tong University Affiliated Sixth People’s Hospital; Shanghai Diabetes Institute; Shanghai Clinical Center for Diabetes; Shanghai Key Clinical Center for Metabolic Diseases; Shanghai Key Laboratory of Diabetes Mellitus, 600 Yishan Road, Shanghai 200233, China; 2 Department of Endocrinology and Metabolism, HuaShan Hospital, Institute of Endocrinology and Diabetology at Fudan University, 12 Wulumuqi Road, Shanghai 200040, China

**Keywords:** Urine albumin creatinine ratio, Carotid intima-media thickness, Carotid plaque, Carotid stenosis, Type 2 diabetes mellitus

## Abstract

**Background:**

Low-grade albuminuria is associated with cardiovascular risk factors and mortality. Our aim was to investigate the association between low-grade albuminuria and carotid atherosclerotic lesions in community-based patients with type 2 diabetes.

**Methods:**

A cross-sectional study was performed in 475 community-based patients with type 2 diabetes (190 males and 285 females) with normal urinary albumin-to-creatinine ratios (UACR) (< 3.5 mg/mmol) from Shanghai, China. The subjects were stratified into tertiles based on UACR levels (the lowest tertile was UACR ≤ 1.19 mg/mmol, and the highest tertile was UACR ≥ 2 mg/mmol). Carotid intima-media thickness (CIMT), carotid atherosclerotic plaque formation and stenosis were assessed and compared among the three groups based on ultrasonography. The urinary albumin excretion rate was determined as the mean of the values obtained from three separate early morning urine samples.

**Results:**

Compared with the subjects with UACR in the lowest tertile, the subjects with UACR in the middle and highest tertiles had greater CIMT values (0.88 ± 0.35 mm, 0.99 ± 0.43 mm and 1.04 ± 0.35 mm, respectively; all p < 0.05) and a higher prevalence of carotid atherosclerotic plaques (25.3%, 39.0% and 46.2%, respectively; all p < 0.05) after adjusting for sex and age. Fully adjusted multiple linear regression and logistic regression analyses revealed that UACR tertiles were significantly associated with elevated CIMT (p = 0.007) and that, compared with the subjects in the first tertile of UACR, those in the second and third tertiles had 1.878- and 2.028-fold risk of carotid plaques, respectively. However, there was no statistical association between UACR tertile and the prevalence of carotid stenosis.

**Conclusions:**

Higher UACR within the normal range was independently associated with early but not late carotid atherosclerotic lesions in community-based patients with type 2 diabetes. Low-grade albuminuria contributes to the risk of carotid atherosclerosis and may be used as an early marker for the detection of atherosclerosis in patients with type 2 diabetes.

## Background

Albuminuria has been extensively recommended as a reliable predictive marker for early detection of diabetic microangiopathy, including diabetic nephropathy, retinopathy and neuropathy [1-3]. It is also reported that albuminuria is closely related with risk of cardiovascular disease and death [4-7], and microalbuminuria is strongly associated with carotid intima-media thickness (CIMT), a surrogate index of general atherosclerosis [8,9]. Recently, a slight elevated urinary albumin-to-creatinine ratio (UACR) level, even below the current cutoff point of microalbuminuria, was also observed to be associated with higher CIMT in general populations and in individuals with diabetes mellitus [10-13]. Furthermore, the Shanghai Changfeng Study demonstrated that a 1-unit increase in the logUACR was associated with a nearly 1.9-fold risk of the presence of carotid plaque in male normotensive and euglycemic Chinese middle-aged and elderly adults; in female adults, this risk was nearly 2.4-fold [12].

However, in patients with type 2 diabetes, the data are not well established with regard to the association between low-grade albuminuria and carotid atherosclerotic lesions, including CIMT, atherosclerotic plaque and stenosis. Therefore, the aim of our study was to comprehensively investigate the relationship between low-grade albuminuria within the normal range (<3.5 mg/mmol) as measured by UACR and carotid atherosclerotic lesions in Chinese community-based patients with type 2 diabetes mellitus.

## Materials and methods

### Subjects and study design

We used the data from our previous study, which aimed to determine the prevalence of diabetic complications in Chinese patients in downtown Shanghai who were diagnosed with type 2 diabetes in 2004 [14]. Briefly, 1039 Chinese patients aged over 30 who were diagnosed with type 2 diabetes were enrolled in the present study. These patients were from twenty residential areas randomly selected from two of downtown Shanghai’s urban areas. Of these, we excluded 564 participants for the following reasons: lack of physical examination and laboratory assessments (21 cases), the presence of kidney disease or other diseases contributing to proteinuria, such as systemic lupus erythematosus (12 cases), 2 of 3 UACR measurements greater than 3.5 mg/mmol (492 cases), and lack of carotid ultrasonography data (39 cases). Four hundred seventy-five subjects, including 190 males and 285 females, were included in the final analysis. The present study was approved by the Ethics Committee of HuaShan Hospital, and written consent was obtained from all participants.

### Physical examination and laboratory measurements

The physical and laboratory examinations used in this study have been described previously [14]. The glomerular filtration rate (GFR) was estimated based on serum creatinine concentration using the simplified MDRD formula: estimated GFR (eGFR) = 186.3× (Serum creatinine)^-1.154^× (age)^-0.203^(×0.742 if female) [15]. The urinary albumin excretion rate was determined as the mean of three separate early morning urine samples obtained over a period of 3 months. UACR was calculated from urinary albumin divided by urinary creatinine based on the screening protocol of the American Diabetes Association [16]; 2 of 3 urinary albumin-creatinine ratios less than 3.5 mg/mmol within a period of 3 months were categorized as normoalbuminuria.

### Ultrasonography measurements

Carotid ultrasonography was performed using a machine with a phased-array transducer (Acuson Sequoia 512, Siemens) and was conducted by certified, proficient sonographers. The ultrasound scanning protocol used in the present study was modified from procedures used in previous studies [17-19]. That is, the sonographers successively recorded and read bilateral images of the common carotid arteries (1 cm proximal to the dilatation of the carotid bulb), the carotid bulb (identified by the loss of the parallel wall present in the common carotid artery), and the internal carotid artery (1 cm distal to the tip of the flow divider that separates the external and internal carotid arteries). The intima-media thickness was the distance between the lumen-intima interface and the media-adventitia interface [20]. CIMT was defined as the mean of the right and left IMTs of the common carotid artery. Plaque within the carotid artery was defined as a localized protrusion of the internal part of the vessel wall into the lumen of 50% of the surrounding IMT value [21]. Carotid stenosis was defined as any degree of narrowing of the carotid arteries by carotid plaques [22].

### Statistical analyses

The data were analyzed using SPSS 15.0 software. For continuous variables, normality was checked. If the data conformed to a normal distribution, variables were given as the mean ± S.D. and one-way ANOVA with LSD was used to determine differences among groups. If the data were not distributed normally, the Kruskal-Wallis test was employed and variables were expressed as the median. Categorical variables were represented either as absolute numbers or as percentages. Chi-squared statistical analysis was utilized to determine differences in categorical variables. Both stepwise forward multiple linear regression and binary logistic regression analyses were performed to examine the association between UACR tertiles and carotid atherosclerotic lesions and to assess the correlations of three parameters of carotid lesions with each other. P <0.05 (two-sided) was considered to be statistically significant.

## Results

### Basal clinical and laboratory characteristics of the subjects

The patients were divided into three groups according to the cutoff points of the UACR tertiles. The lowest tertile was UACR ≤1.19, and the highest tertile was UACR ≥ 2 mg/mmol. Table 1 shows the clinical and biochemical parameters of the studied subjects according to the UACR tertile groups. Age, duration of diabetes, body mass index (BMI), prevalence of hypertension, diastolic blood pressure (DBP), 2 h postprandial plasma glucose (2 h PPG), and hemoglobin A1C (HbA1c) progressively increased from the lowest UACR tertile to the highest tertile (all p < 0.05) even after adjustment for age and sex. Systolic blood pressure (SBP), fasting plasma glucose (FPG) and eGFR were also significantly different among the three groups when age and sex were examined (all p < 0.05). Other studied indicators were not significantly different among the three groups.

**Table 1:** Clinical characteristics of the subjects

**Variables**	**1**^**st **^**tertile (n = 158)**	**2**^**nd **^**tertile (n = 159)**	**3**^**rd **^**tertile (n = 158)**	***p *value**	*p value
UACR range (mg/mmol)	≤1.19	1.20-1.99	≥2.00	-	-
AGE (y)	63 ± 11	65 ± 10	68 ± 9	<0.001	<0.001
Male, n (%)	69(43.7)	64(40.3)	57(36.1)	0.386	0.129
Duration of diabetes (y)	6 ± 6	7 ± 7	9 ± 8	0.002	0.032
Smoking, n (%)	40(25.3)	38(23.9)	26(16.5)	0.123	0.886
Alcohol, n (%)	18(11.4)	22(13.8)	21(13.3)	0.792	0.318
BMI (kg/m^2^)	23.95 ± 2.89	24.94 ± 3.74	25.03 ± 3.38	0.008	0.002
WHR	0.87 ± 0.06	0.88 ± 0.08	0.88 ± 0.06	0.337	0.232
Hypertension, n (%)	85(53.8)	89(56.0)	113(71.5)	0.002	0.002
*SBP (mmHg)	135(115–150)	120(110–140)	130(120–140)	<0.001	<0.001
DBP (mmHg)	80 ± 13	81 ± 11	84 ± 11	0.012	0.001
*FPG (mmol/L)	7.4(6.5-8.5)	7.8(6.7-9.8)	7.7(6.6-9.9)	0.049	0.025
*2 h PPG (mmol/L)	12.1(9.7-16.9)	13.9(10.9-17.1)	14.1(9.9-19.6)	0.032	0.028
HbA1c (%)	6.82 ± 1.39	6.99 ± 1.50	7.19 ± 1.53	0.089	0.026
FIN (uIU/mL)	16.57 ± 32.37	14.43 ± 10.12	15.06 ± 12.33	0.645	0.531
2 h IN (uIU/mL)	52.50 ± 47.08	50.07 ± 41.87	58.62 ± 65.39	0.331	0.946
HOMA-IR	5.34 ± 7.20	5.59 ± 4.48	5.57 ± 4.59	0.91	0.555
BUN (mmol/L)	5.98 ± 1.47	5.93 ± 1.30	6.11 ± 1.56	0.531	0.804
Scr (umol/L)	69.22 ± 15.70	65.48 ± 16.00	66.50 ± 16.73	0.102	0.069
UA (mmol/L)	0.30 ± 0.08	0.29 ± 0.08	0.28 ± 0.07	0.061	0.66
eGFR (ml/min/1.73 m2)	95.11 ± 21.27	100.10 ± 24.17	96.65 ± 23.83	0.146	0.01
TC (mmol/L)	5.13 ± 0.87	5.45 ± 1.08	5.33 ± 1.06	0.015	0.201
*TG (mmol/L)	1.63(1.03-2.26)	1.61(1.10-2.24)	1.64(1.08-2.07)	0.941	0.916
HDL-C (mmol/L)	1.24 ± 0.33	1.33 ± 0.39	1.28 ± 0.36	0.716	0.689
*LDL-C (mmol/L)	2.90(2.50-3.30)	3.00(2.40-3.70)	3.00(2.50-3.60)	0.515	0.935

Values are presented as mean ± S.D, median with interquartile range, or percentage. *Non-normal distribution of continuous variables.

P value: The p-values were not adjusted for age and sex for the trend.

*P value: The p-values were adjusted for age and sex for the trend.

### Comparison of carotid atherosclerotic lesions among tertile groups of UACR

**Figure 1:**
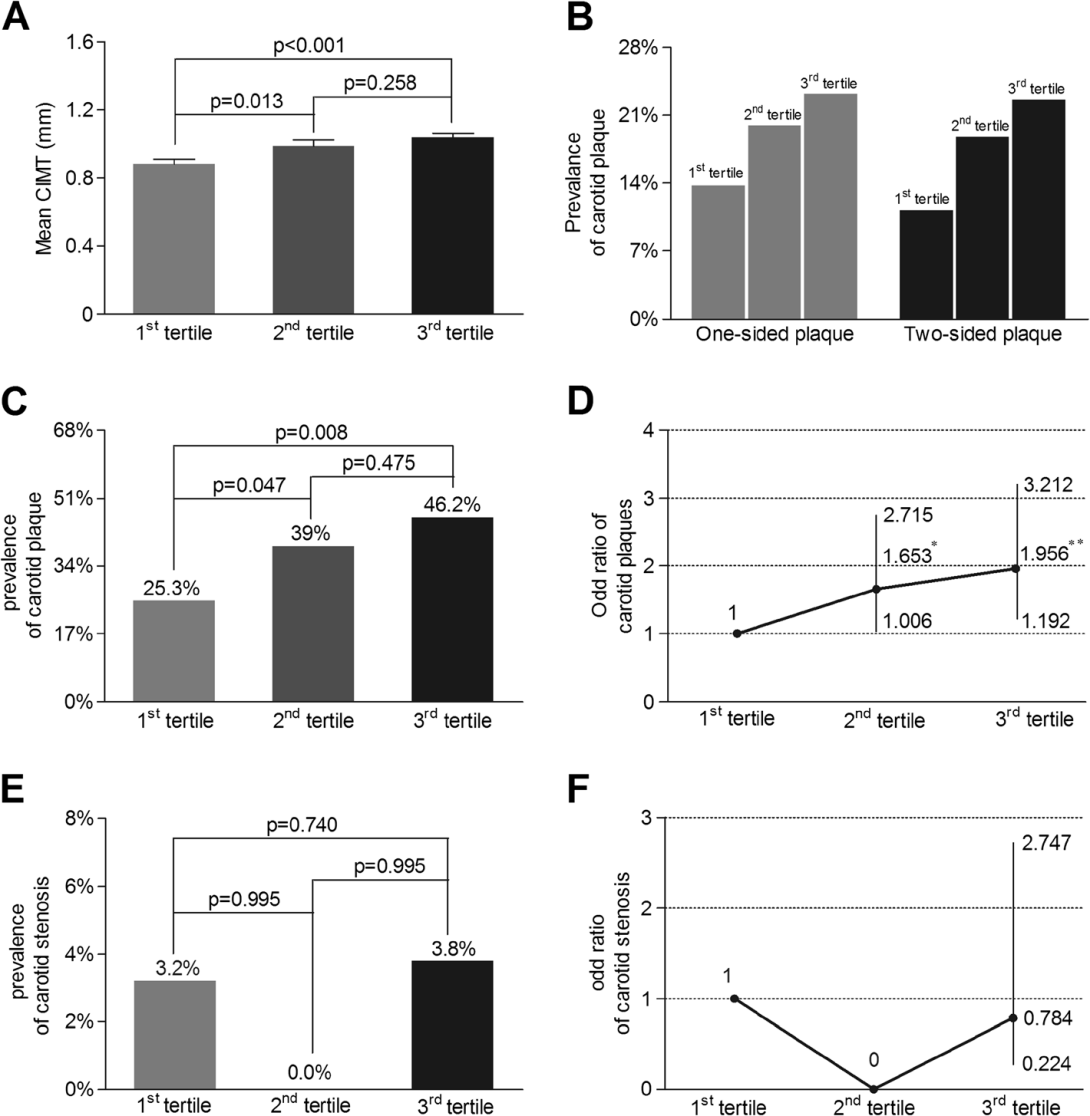
**Comparison of carotid atherosclerotic lesions among UACR tertile groups after adjusting for age and sex. (A)** Comparison of mean CIMT among the three groups. The p-value for the three-group comparison was 0.01. **(B)** The distribution of carotid atherosclerotic plaque among the three groups. The p-value for the three-group comparison was 0.354 for one-sided plaque and 0.158 for two-sided plaque **(C)** Comparisons of the prevalence of carotid atherosclerotic plaque among three groups. The p-value for three group comparison was 0.026. **(D)** Odds ratio of carotid plaque for the 2^nd^ and 3^rd^ UACR tertile subjects in comparison to the 1^st^ UACR tertile subjects. The bar represents the 95% confidence interval. Compared with the 1^st^ tertile, *p = 0.047 and **p = 0.008, respectively. **(E)** Comparison of the prevalence of carotid stenosis among the three groups. The p-value for the three-group comparison was 0.954. **(F)** Odds ratio of carotid plaque for the 2^nd^ and 3^rd^ UACR tertile subjects in comparison to the 1^st^ UACR tertile subjects. The bar represents the 95% confidence interval.

A comparison of carotid atherosclerotic lesions among the UACR tertile groups after adjustment for age and sex is shown in Figure 1. Compared with the subjects in the first UACR tertile, those in the second and third tertiles had significantly higher values of CIMT (0.99 ± 0.43 mm and 1.04 ± 0.35 mm vs. 0.88 ± 0.35 mm, respectively) (Figure 1A) and a higher prevalence of carotid plaques (Figure 1C), whereas there was no significant difference in the prevalence of carotid stenosis among the three groups (Figure 1E). The distributions of both one-sided plaques (13.9%, 20.1% and 23.4%, respectively; p = 0.093) and two-sided plaques (11.4%, 18.9% and 22.8%, respectively; p = 0.026) showed a steady rising trend across the UACR tertiles. However, there were no significant differences in these parameters after adjustment for age and sex (p = 0.354 and p = 0.158, respectively) (Figure 1B). In addition, a significant increase in the odds ratio of carotid plaque was observed in the middle and highest tertiles relative to the lowest one (p = 0.047 and p = 0.008, respectively) (Figure 1D), but no significant differences in the odds ratio of carotid stenosis were found among the three groups (Figure 1F).

### Comparison of carotid atherosclerotic lesions stratified by sex and age in each UACR tertile group

Analyses of carotid atherosclerotic lesions stratified by sex and age in each of the UACR tertile groups are shown in Table 2. The data in the table demonstrate that significant sex-related differences in the mean CIMT and in the prevalence of carotid plaque existed only in the third UACR tertile. In addition, a remarkable increase in the mean CIMT and in the prevalence of carotid plaque between middle-aged (age < 65) and older (age ≥ 65) patients was observed in each tertile group. In contrast, there were neither sex-related nor age-related significant differences in the prevalence of carotid stenosis in any of the tertile groups.

**Table 2:** Comparison of carotid atherosclerotic lesions stratified by sex and age in each UACR tertile group

		**Mean CIMT**		**Carotid plaque**		**Carotid stenosis**	
		**Value (mm)**	**p value**	**Prevalence (%)**	**p value**	**Prevalence (%)**	**p value**
**Sex**							
1^st^ tertile	Male	0.94 ± 0.42	0.1	33.30%	0.053	4.30%	0.472
	Female	0.84 ± 0.28		19.10%		2.30%	
2^nd^ tertile	Male	1.01 ± 0.48	0.634	39.10%	0.925	0.00%	N/A
	Female	0.97 ± 0.39		38.90%		0.00%	
3^rd^ tertile	Male	1.12 ± 0.37	0.045	36.80%	0.04	5.30%	0.774
	Female	0.99 ± 0.33		51.50%		3.00%	
**Age**							
1^st^ tertile	Age < 65	0.80 ± 0.29	0.001	17.00%	0.009	3.40%	0.832
	Age ≥ 65	0.99 ± 0.39		35.70%		2.90%	
2^nd^ tertile	Age < 65	0.94 ± 0.45	0.062	25.40%	0.005	0.00%	N/A
	Age ≥ 65	1.02 ± 0.42		47.90%		0.00%	
3^rd^ tertile	Age < 65	0.91 ± 0.33	0.002	32.00%	0.013	0.00%	0.997
	Age ≥ 65	1.09 ± 0.34		52.80%		5.60%	

P value: The p values were adjusted for either sex or age for the trend.

### Association between carotid atherosclerotic lesions and UACR tertiles

Table 3 shows the association of the UACR tertiles with the value of CIMT and the presence of carotid plaques based on both multiple linear regression and binary logistic regression analyses. After controlling for age, sex, smoking, drinking, duration of diabetes and hypertension (ModelI), a significant association was observed between increased UACR tertile and increased CIMT (p = 0.011) but not between increased UACR tertile and the presence of carotid plaques (p = 0.099). However, after adding several clinical indicators (Model II), both CIMT and carotid plaques were positively associated with increased UACR tertile (p = 0.003 and 0.017, respectively). Moreover, compared with the subjects in the first UACR tertile, those in the second and third tertiles had 1.876- and 2.016-fold risk of carotid plaques, respectively. In Model III, increased UACR tertile remained a significant predictor of both CIMT and carotid plaques even after controlling for various clinical risk factors (p = 0.007 and 0.018, respectively). Accordingly, the patients in the second and third UACR tertiles had 1.878- and 2.028-fold risk of carotid plaque, respectively, relative to those in the lowest tertile. However, no significant association was found between UACR tertile and carotid stenosis whether or not adjustment for clinical and biochemical parameters were performed (Additional file 1: Table S1).

**Table 3:** Association of UACR tertile with CIMT and carotid plaques by linear regression and binary logistic regression

	**Mean CIMT**			**Carotid plaque**			
	**β**	**95% CI**	**P value**	**1**^**st **^**tertile**	**2**^**nd **^**tertile**	**3**^**rd **^**tertile**	**P value**
				**OR**	**OR (95% CI)**	**OR (95% CI)**	
Model I	0.055	0.013-0.097	0.011	1	1.608 (0.971-2.662)	1.662 (0.995-2.775)	0.099
Model II	0.065	0.022-0.107	0.003	1	1.876 (1.118-3.148)	2.016 (1.199-3.391)	0.017
Model III	0.058	0.016-0.100	0.007	1	1.878 (1.116-3.161)	2.028 (1.195-3.439)	0.018

Model I: Adjusted for age, sex, smoking, alcohol, duration of diabetes and hypertension.

Model II: Adjusted for age, sex, smoking, alcohol, duration of diabetes, hypertension, BMI, WHR, SBP, DBP, and eGFR.

Model III: Adjusted for age, sex, smoking, alcohol, duration of diabetes, hypertension, BMI, WHR, SBP, DBP, eGFR, FPG, 2 h PPG, HbA1c, FIN, 2hIN, HOMA-IR, BUN, Scr, UA, TG, TC, and LDL-C.

### The correlations of parameters of carotid lesions with each other

The correlations of parameters of carotid lesions with each other were analysed by multiple linear regression and by binary logistic regression. The fully adjusted regressions indicated that CIMT correlated with carotid plaque and stenosis, respectively, although carotid plaque did not correlate with carotid stenosis (Additional file 2: Table S2).

## Discussion

In the present study, we found that higher UACR within the normal range was associated with elevated CIMT and with higher incidence of carotid plaque but not with carotid stenosis, even after adjusting for traditional cardiovascular disease risk factors and eGFR in Chinese community-based patients with T2DM. To the best of our knowledge, this is the first time the relationship between low-grade albuminuria and carotid atherosclerotic lesions has been systematically studied in Chinese patients with type 2 diabetes. The association of microalbuminuria with cardiovascular disease has been well established in a number of populations, including diabetics, hypertensive patients and general subjects [23-26], but not until recently have several studies reported that cardiovascular morbidity and mortality are increased even in patients with much lower UACR levels. For example, the HOPE study demonstrated that UACR above 0.58 mg/mmol, a value well below the cutoff for microalbuminuria, had remarkable correlation with cardiovascular events and that every 0.4 mg/mmol increment in UACR was associated with a 5.9% increase in the risk of occurrence of a major cardiovascular event [5]. The Framingham Heart Study also revealed that subjects with UACR values at or above 3.9 mg/g (equivalent to 0.52 mg/mmol) for men and 7.5 mg/g (equivalent to 0.99 mg/mmol) for women were associated with a 3-fold risk of developing cardiovascular diseases in comparison to patients with UACR below these levels [27]. Likewise, the Strong Heart Study indicated that albuminuria levels below the threshold definition of microalbuminuria can predict cardiovascular diseases in middle-aged and elderly American Indians [28].

The foregoing findings indicate that further exploration of the association between the normal range of UACR and the occurrence of cardiovascular disease is of great importance for the early detection and intervention of such disease. Although CIMT has been widely adopted as an early marker of cardiovascular disease [29], it does not well predict the absence, extent, severity or prognosis of coronary artery disease, which carotid plaque and stenosis do [30-33]. We therefore comprehensively studied the association between UACR within the normal range and carotid atherosclerotic lesions, including CIMT, carotid plaque and stenosis.

Consistent with our results, Huang et al. [11] and Kweon et al. [13] also showed that higher normal ranges of UACR are positively and independently associated with CIMT in patients with diabetes and in the general population, respectively. In the present study, men had higher CIMT than women in each UACR tertile, but a significant difference between men and women existed only in the highest UACR tertile, in which the mean age was 68. Similar findings were also reported by Bo et al., who found a gender difference in CIMT only in a group in which the mean age was greater than 69 years [34]. Another main finding of our study was that patients 65 years of age or older also had higher CIMT than patients below 65 years of age, a result that is in keeping with other studies [35,36].

Consistent with the results of the Shanghai Changfeng Study [12], we found that the odds ratio of carotid plaques increased steadily across UACR tertiles in patients with type 2 diabetes. Lee et al. also demonstrated that compared with low normoalbuminuria subjects (UACR < 15.0 mg/g, equivalent to 1.99 mg/mmol), community-dwelling Koreans with high normoalbuminuria (UACR > 15.0 mg/g, equivalent to 1.99 mg/mmol) had significantly higher risk of carotid plaque [37].

Importantly, our study demonstrated that there was no significant association between normal UACR and carotid stenosis even after adjustment for other variables. This may be due to the lower prevalence of carotid stenosis compared to carotid plaque [38,39] and the fact that it represents a later stage in the atherosclerotic process [40]. To our knowledge, this is the first time the relationship between UACR in the high normal range and carotid stenosis has been investigated, although some authors have reported that microalbuminuria correlates with the prevalence and severity of coronary artery stenosis [41].

Similar to our previous and other studies [22,42-44], we found that older patients (age ≥ 65) had a higher mean CIMT and prevalence of carotid plaque compared with younger patients (age < 65) in each UACR tertile group. Gender difference in carotid atherosclerosis has been confirmed in general population and patients with diabetes [34,35,45], but in the present study, sex-related differences in the mean CIMT and in the prevalence of carotid plaque existed only in the highest UACR tertile group. This can be explained by the fact that age in the highest UACR tertile group was significantly older than in the first and second UACR tertile groups. This finding was consistent with a previous study [35], which found that sex difference in CIMT was not significant in young population and gradually increased with age [35]. Unlike CIMT and carotid plaque, there were neither sex-related nor age-related significant differences in the prevalence of carotid stenosis in any of the tertile groups, which may be due to low prevalence of carotid stenosis and relatively small samples in the present study.

Lastly, consistent with the results of previous studies [46-48], our study demonstrated that three parameters of carotid lesions (CIMT, atherosclerotic plaque, and stenosis) generally correlate well with each other. Bonithon et al. [49] reported that the odds ratio for having at least one plaque associated with a 0.10 mm increase in CIMT was 1.18 in the EVA study. Gnasso et al. [50] showed that CIMT was strongly and significantly associated with the presence of plaques and/or stenosis in the carotid arteries after adjustment for coronary heart disease risk factors. The Aging Vascular Study [51], the Tromsø Study [52], the San Daniele Project [53] and a study of health in Pomerania [54] also confirmed that CIMT independently predicts incident carotid plaque formation in a prospective manner.

There is a remaining controversial issue concerning the independent correlation between albuminuria and atherosclerosis. Ninomiya et al. [55] and Sung et al. [10] noted that elevated CIMT was associated with increased albuminuria after excluding other compound variables, whereas Ishimura et al. [56] and Ito et al. [57] reported that significant association between these two parameters was lost after adjustment for traditional risk factors such as blood pressure and waist-hip ratio. Our results showed that the statistical significance between UACR tertiles and carotid plaque was lost after application of the binary logistic regression analysis in Model I. However, after adding additional risk factors (Model II and Model III), UACR tertile was positively associated with carotid plaque, suggesting that these additional variables may act as compounding factors. When we excluded them, the association between UACR and carotid plaque was obvious. In studies involving patients with diabetes, the duration of diabetes may be the primary cause of discrepancies in the results. For example, Shin et al. [58] reported that microalbuminuria was not related to CIMT in newly diagnosed patients with type 2 diabetes. The median duration of diabetes in our patients and in the study of Huang et al. [11] was 5–7 years, while the patients in Shin’s study had a shorter duration of diabetes of approximately 1 year. Because duration of diabetes is closely related to CIMT in patients with type 2 diabetes [59], studies of patients with different durations of diabetes appear to yield different results. Furthermore, the basic characteristics of the patients in the studies (their ethnic diversity, for example) as well as the use of different methods to measure carotid atherosclerotic lesions could also lead to discrepancies.

The mechanism or mechanisms by which albuminuria increases atherosclerosis are not well-elucidated, but endothelial dysfunction [60-63] and low-grade chronic inflammation [64,65] may be responsible for common underlying mechanisms. In addition, synergistic effects of albuminuria in combination with cardiovascular risk factors such as hypertension and dyslipidemia may also partly explain why albuminuria even at levels below the microalbuminuria threshold remains a strongly independent indictor for atherosclerosis. Further studies will be needed to clarify the exact relationship between these factors.

### Limitations

Our study adds powerful clinical evidence supporting the hypothesis that risk of cardiovascular diseases may begin to increase at relatively low UACR levels. However, our study has several limitations that should be noted. First, the number of patients studied was relatively small. Thus, prospective studies of a larger sample should be conducted to verify the relationship between low-grade albuminuria and the presence of carotid atherosclerotic lesions. Second, due to the relatively small sample size, we did not analyze the subjects according to sex-specific UACR tertiles; instead, we studied sex-related differences in each UACR tertile subgroup after adjustment for age. Third, we did not consider the patients’ medications.

## Conclusions

In conclusion, this study demonstrated that higher UACR within the normal range was independently associated with early atherosclerotic lesions, including CIMT and carotid plaque, but not with late atherosclerotic lesions manifested by carotid stenosis, after adjustment for conventional risk factors in patients with type 2 diabetes. The results imply that low-grade albuminuria contributes to the risk of atherosclerosis and that it may represent an early marker for the detection of atherosclerosis in patients with type 2 diabetes.

## Abbreviations

UACR: Urinary albumin-to-creatinine ratio; CIMT: Carotid intima-media thickness; GFR: Glomerular filtration rate; eGFR: Estimated glomerular filtration rate; T2DM: Type 2 diabetes mellitus; BMI: Body mass index; WHR: Waist-hip ratio; SBP: Systolic blood pressure; DBP: Diastolic blood pressure; FPG: Fasting plasma glucose; 2 h PPG: 2 h Postprandial plasma glucose; HbA1c: Hemoglobin A1C; FIN: Fasting insulin; 2 h IN: 2 h Insulin; HOMA-IR: Homeostatic model assessment of insulin resistance; BUN: Blood urea nitrogen; Scr: Serum creatinine; UA: Uric acid; TC: Total cholesterol; TG: Triglyceride; HDL-C: High-density lipoprotein cholesterol; LDL-C: Low-density lipoprotein cholesterol; 95% CI: 95% confidence interval.

## Competing interests

The authors declare that they have no competing interests.

## Authors’ contributions

LX Li designed the study, supervised the work, and reviewed and edited the manuscript. MF Li and YF Tu researched data, performed statistical analysis and wrote the manuscript. JX Lu, XH Dong, LB Yu, R Zhang, YQ Bao, WP Jia and RM Hu researched data and reviewed the manuscript.

## Supplementary Material

Additional file 1: Table S1 Association of UACR tertile with carotid stenosis by binary logistic regression. Click here for file

Additional file 2: Table S2 The correlation among parameters of carotid lesions by logistic regression. Click here for file
