# Supplementary material for: Low-grade albuminuria is associated with early but not late carotid atherosclerotic lesions in community-based patients with type 2 diabetes
Source: Cardiovasc Diabetol. 2013 Jul 24;12:110. doi: 10.1186/1475-2840-12-110 (PMC3725174; doi:10.1186/1475-2840-12-110)
Supplement: Additional file 1: Table S1 — Association of UACR tertile with carotid stenosis by binary logistic regression. [file 1475-2840-12-110-S1.doc]

**Additional file 1**

**Supplementary table 1** **Association of UACR tertile with carotid stenosis by binary logistic regression**

|  | Comparison between two groups | OR (95% CI) | p value |
| --- | --- | --- | --- |
| Model Ⅰ | Tertile 2 vs. Tertile 1 | 0 | 0.995 |
|  | Tertile 3 vs. Tertile 1 | 0.928(0.251-3.429) | 0.910 |
|  | Tertile 3 vs. Tertile 2 | 0 | 0.995 |
| Model Ⅱ | Tertile 2 vs. Tertile 1 | 0 | 0.995 |
|  | Tertile 3 vs. Tertile 1 | 1.816(0.362-9.108) | 0.468 |
|  | Tertile 3 vs. Tertile 2 | 0 | 0.995 |
| Model Ⅲ | Tertile 2 vs. Tertile 1 | 0 | 0.995 |
|  | Tertile 3 vs. Tertile 1 | 1.416(0.182-11.012) | 0.456 |
|  | Tertile 3 vs. Tertile 2 | 0 | 0.995 |

Model Ⅰ: Adjusted for age, sex, smoking, alcohol, duration of diabetes and hypertension.

Model Ⅱ: Adjusted for age, sex, smoking, alcohol, duration of diabetes, hypertension, BMI, WHR, SBP, DBP, and eGFR.

Model Ⅲ: Adjusted for age, sex, smoking, alcohol, duration of diabetes, hypertension, BMI, WHR, SBP, DBP, eGFR, FPG, 2h PPG, HbA1c, FIN, 2hIN, HOMA-IR, BUN, Scr, UA, TG, TC, and LDL-C.
