# Supplementary material for: Low-grade albuminuria is associated with early but not late carotid atherosclerotic lesions in community-based patients with type 2 diabetes
Source: Cardiovasc Diabetol. 2013 Jul 24;12:110. doi: 10.1186/1475-2840-12-110 (PMC3725174; doi:10.1186/1475-2840-12-110)
Supplement: Additional file 2: Table S2 — The correlation among parameters of carotid lesions by logistic regression. [file 1475-2840-12-110-S2.doc]

**Additional file 2: Supplementary table 2 The correlation among parameters of carotid lesions by logistic regression**

|  | Dependent variable | Independent variables | β or OR | 95%CI | P value |
| --- | --- | --- | --- | --- | --- |
| **Model I** |  |  |  |  |  |
|  | CIMT | Carotid plaque | 0.145 | 0.074-0.216 | <0.001 |
|  |  | Carotid stenosis | 0.303 | 0.086-0.520 | 0.006 |
|  | Carotid plaque | CIMT | 3.05 | 1.732-5.371 | <0.001 |
|  |  | Carotid stenosis | 1.621 | 0.391-6.726 | 0.506 |
|  | Carotid stenosis | CIMT | 4.403 | 1.623-11.942 | 0.004 |
|  |  | Carotid plaque | 1.883 | 0.503-7.051 | 0.348 |
| **Model II** |  |  |  |  |  |
|  | CIMT | Carotid plaque | 0.152 | 0.081-0.223 | <0.001 |
|  |  | Carotid stenosis | 0.211 | -0.017-0.438 | 0.069 |
|  | Carotid plaque | CIMT | 3.254 | 1.776-5.959 | <0.001 |
|  |  | Carotid stenosis | 2.397 | 0.494-11.644 | 0.278 |
|  | Carotid stenosis | CIMT | 3.685 | 1.227-11.073 | 0.020 |
|  |  | Carotid plaque | 2.476 | 0.498-12.308 | 0.268 |
| **Model III** |  |  |  |  |  |
|  | CIMT | Carotid plaque | 0.127 | 0.057-0.197 | <0.001 |
|  |  | Carotid stenosis | 0.228 | 0.060-0.503 | 0.013 |
|  | Carotid plaque | CIMT | 3.256 | 1.776-5.971 | <0.001 |
|  |  | Carotid stenosis | 2.825 | 0.564-14.162 | 0.207 |
|  | Carotid stenosis | CIMT | 8.223 | 2.271-29.782 | 0.001 |
|  |  | Carotid plaque | 4.604 | 0.699-52.557 | 0.102 |

Model Ⅰ: Adjusted for age, sex, smoking, alcohol, duration of diabetes, hypertension and corresponding independent variables in the table.

Model Ⅱ: Adjusted for age, sex, smoking, alcohol, duration of diabetes, hypertension, BMI, WHR, SBP, DBP, eGFR and corresponding independent variables in the table.

Model Ⅲ: Adjusted for age, sex, smoking, alcohol, duration of diabetes, hypertension, BMI, WHR, SBP, DBP, eGFR, FPG, 2h PPG, HbA1c, FIN, 2hIN, HOMA-IR, BUN, Scr, UA, TG, TC, LDL-C and corresponding independent variables in the table.
